# Wireless access to a pharmaceutical database: A demonstrator for data driven Wireless Application Protocol applications in medical information processing

**DOI:** 10.2196/jmir.3.1.e4

**Published:** 2001-03-17

**Authors:** Michael Schacht Hansen, Jens Dørup

**Affiliations:** ^1^Section for Health InformaticsInstitute of BiostatisticsUniversity of AarhusDenmark

**Keywords:** Medical Informatics Applications, Database Management Systems, Dictionaries, Pharmaceutical, Wireless Application Protocol, Open source software

## Abstract

**Background:**

The Wireless Application Protocol technology implemented in newer mobile phones has built-in facilities for handling much of the information processing needed in clinical work.

**Objectives:**

To test a practical approach we ported a relational database of the Danish pharmaceutical catalogue to Wireless Application Protocol using open source freeware at all steps.

**Methods:**

We used Apache 1.3 web software on a Linux server. Data containing the Danish pharmaceutical catalogue were imported from an ASCII file into a MySQL 3.22.32 database using a Practical Extraction and Report Language script for easy update of the database. Data were distributed in 35 interrelated tables. Each pharmaceutical brand name was given its own card with links to general information about the drug, active substances, contraindications etc. Access was available through 1) browsing therapeutic groups and 2) searching for a brand name. The database interface was programmed in the server-side scripting language PHP3.

**Results:**

A free, open source Wireless Application Protocol gateway to a pharmaceutical catalogue was established to allow dial-in access independent of commercial Wireless Application Protocol service providers. The application was tested on the Nokia 7110 and Ericsson R320s cellular phones.

**Conclusions:**

We have demonstrated that Wireless Application Protocol-based access to a dynamic clinical database can be established using open source freeware. The project opens perspectives for a further integration of Wireless Application Protocol phone functions in clinical information processing: Global System for Mobile communication telephony for bilateral communication, asynchronous unilateral communication via e-mail and Short Message Service, built-in calculator, calendar, personal organizer, phone number catalogue and Dictaphone function via answering machine technology. An independent Wireless Application Protocol gateway may be placed within hospital firewalls, which may be an advantage with respect to security. However, if Wireless Application Protocol phones are to become effective tools for physicians, special attention must be paid to the limitations of the devices. Input tools of Wireless Application Protocol phones should be improved, for instance by increased use of speech control.

## Introduction

The Global System for Mobile communication (GSM) digital wireless network that is used to transmit audio communication in cellular phones may also be used to transmit data at rates that are typically limited to 9600 bits/s. However, for access to the Internet a mobile phone needs connection to a computing device, i.e. either a portable or stationary computer or a Personal Digital Assistant (PDA) with an appropriate interface connection. The Wireless Application Protocol (WAP) is a specification for a communication protocol used to standardize the way wireless devices, such as cellular telephones and radio transceivers, can be used for Internet access, including e-mail and the World Wide Web. The aim of using a standard protocol is to enable devices and service systems that use WAP to operate together. The advantage of WAP phones is that connection to the Internet can be obtained using a modem, a small computer, and a dedicated browser all of which are built into the WAP device. On the other hand, the small screen size, keyboard size, lack of pointing device and especially the low bandwidth made it necessary to develop a standard for design of web pages aimed at WAP devices and a modified markup language, the Wireless Markup Language (WML), had to be developed, taking the limitations of the device into consideration. Cellular phones using the WAP for access to the Internet comprise potentials for assisting in handling many clinical information needs [1].

- Conventional GSM telephony for synchronous, two-way voice telephony
- Asynchronous unilateral communication via e-mail and Short Message Service (SMS)
- Dictaphone function using answering machine technology or built-in speech message facilities
- Built-in calendar and personal organizer functions
- Phone number catalogue and other smaller databases built into the device
- Calculator and other dedicated built-in applications

In addition WAP technology allows access to databases on Internet servers - e.g. pharmaceutical information, laboratory data, educational materials, and access can be gained to Internet based Electronic Patient Records [2]. Reference materials (pocket manuals) are often used by physicians in the daily work, but printed reference books are rarely updated and may thus become outdated. Many doctors carry some sort of paging or communications device like a PDA with varying capacity to store clinical databases. There are a number of advantages to be gained by incorporating references manuals and other clinical information into handheld devices through the WAP standard [3]. This would allow easy access to several reference manuals through a single device. Manuals would be updated centrally and dynamically. Although many of the functions mentioned are already available in today's cellular phones, they have only been exploited only to a limited extent. This paper describes our first experiences with porting a pharmaceutical database to a WAP accessible database, involving the following steps:

- a) A pharmaceutical relational database was interfaced with server side scripting and deployed to a WAP device
- b) The information should be formatted in a way suited for small handheld devices
- c) The project was implemented using a standard personal computer without purchase of any new software

## Methods

### Web Server

Establishing a data-driven online resource available to WAP devices requires a modified web server, with a database engine and a programming interface to the database. If the server needs to work as a dial-in interface for the WAP device, a WAP gateway must also be established. All of these features were implemented using free, open source software. Documents served from a web server are associated with a Multi-Purpose Internet Mail Extension (MIME) type. The MIME type is needed by the browser to determine how the file should be processed (e.g. rendered like a normal hypertext markup language (HTML) file or handled by a helper application). The file types used for WAP devices have a new set of MIME types (Table 1) unknown to most web servers and the web server must have these types added.

**Table 1:** WAP MIME types

*MIME type*	File extension	Content
Text/vnd.wap.wml	.wml	WML source code
Application/vnd.wap.wmlc	.wmlc	Compiled WML
Text/vnd.wap.wmlscript	.wmls	WML script source code
Application/vnd.wap.wmlscriptc	.wmlsc	Compiled WML script
image/vnd.wap.wbmp	.wbmp	Wireless bitmap

We used an *Apache 1.3* web server installed on a Linux server. The MIME types were registered by adding the lines shown in (Table 1) to the configuration file "httpd.conf":


AddType text/vnd.wap.wml .wml
AddType application/vnd.wap.wmlc .wmlc
AddType text/vnd.wap.wmlscript .wmls
AddType application/vnd.wap.wmlcscriptc .wmlsc
AddType image/vnd.wap.wbmp .wbmp
                


### Database

Data containing the Danish pharmaceutical catalogue was imported from an ASCII file received every two weeks from the Danish Medical Association. Data was distributed in 35 interrelated tables with easy access to the hierarchy in the pharmaceutical directory, facilitating browsing through the pharmaceutical classes. The database structure also facilitated search for specific brand names or active substances. Import into a MySQL 3.22.32 database was done using a dedicated Practical Extraction and Report Language (Perl) script designed for easy update of the database. The program structure was designed around the brand names. Each brand name was given its own WML page (card) with links to general information about the drug, active substances, contraindications etc. Access to these cards was available through browsing the therapeutic groups or searching for a specific brand name. The text entry was made as simple as possible. Typically only the first three characters of the brand name need to be entered before activating the search.

### Programming

**Figure 1:**
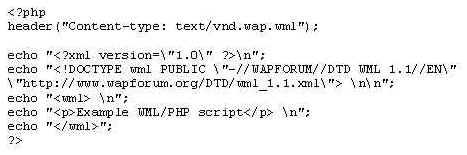
An example of the code to be entered in the header of the WML document for the web

A server-side scripting layer was used to interface the database. The scripting layer is used to a) send SQL queries to the database and b) format the data from the database as WML for interpretation by the WAP gateway. The database interface was programmed in the server-side scripting language PHP3. PHP is designed as a scripting language embedded in HTML and it is designed to generate HTML. To ensure that the content returned by the script was WML the document MIME type was sent explicitly with the "header" function. An example of a PHP script that returns a WML page is shown in Figure 1.

This example does not send any queries to the database, but it illustrates how http headers can be formed with the correct MIME type using PHP. Database queries were handled through the structured query language (SQL) access to the database and the contents of the database were sent to the WAP enabled device. The choice of scripting language is somewhat arbitrary. Other popular scripting languages like Active Server Pages (ASP) or Perl could also have been used. The communication between cellular phone and database could also have been implemented through an executable application on the web server (e.g. C/C++ programming). However, the overhead involved in starting a process for each database request, makes such a solution less feasible. Regardless of the implementation strategy, special care should be taken to ensure that the content-type header field is formed correctly.

### Dataflow

**Figure 2:**
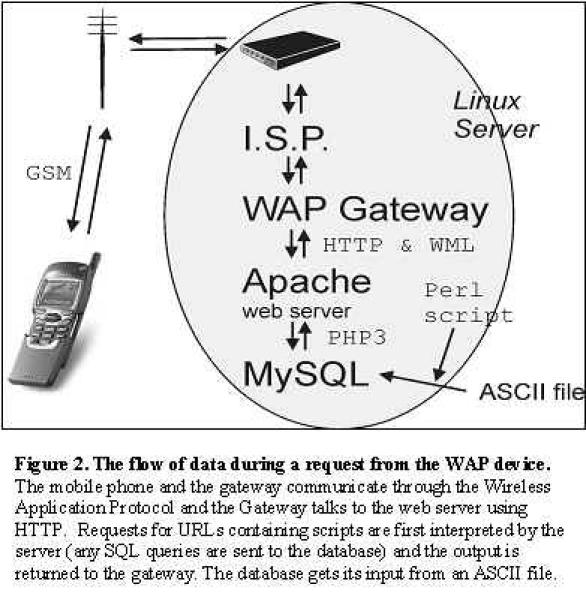
The flow of data during a request from the WAP device

The communication between a handheld device and a database passes through several different layers and different communication protocols are used (Figure 2). The individual layers have restrictions some of which are crucial to the implementation of the WAP application.

The handheld device connects to an Internet Service Provider (ISP) with a standard Point to Point protocol (like connecting to the Internet with a standard modem)w. The ISP is in contact with a WAP gateway; the ISP often provides both the Internet access and WAP gateway. The gateway may be public and provided by one of the mobile telecommunication companies. (See a list of public gateways at 4. www.wapdrive.com. WAP Gateways around the world. www.wapdrive.com/DOCS/wap_gateway/index.html) [4] or it may be private (see below). The role of the ISP is to transmit data between the handheld device and the gateway. The gateway sends requests from the phone to web-servers on the Internet and it encodes the results received from the web-servers to a more compact form to facilitate the communication across the low bandwidth connection. The encoded data is sent to the handheld device using the WAP. The amount of data that can be sent to the handheld device depends on the device. The Nokia 7110 has a limitation of 1397 bytes in the compressed form sent by the gateway [5]. An uncompressed WML document should be kept below 1500 bytes to ensure that handheld devices can receive it. When the handheld device sends a request for a Uniform Resource Locator (URL), the gateway passes the request to the web-server using the standard http-protocol. The web-server handles the requests as it would a normal request for a web page. However, if the requested URL is a WML document the request is returned to the gateway for further processing. If the URL refers to a script (in this case a PHP script), the PHP interpreter will process the script (handle database queries, format the output and return it to the gateway). The gateway will subsequently encode and compress the data for transmission with the WAP protocol.

### WAP Gateway

A WAP Gateway was established for direct dial-in access to the pharmaceutical catalogue. A free, and open source gateway was downloaded from www.kannel.org [6] and installed on a Linux server. The gateway is still being developed and the latest stable version is 0.11.2 (September 29th 2000). The gateway relies on an Extensible Markup Language (XML) parser to interpret the WML pages and the Linux server should have the library: libxml-1.8.7 or higher installed to compile the gateway. For dial-in, a modem (ISDN or analogue) was connected and controlled by dial-in software on the server.

### Phone set-up

The WAP enabled phone must be configured to access the appropriate gateway. Phone number, username and password (for the dial-in connection) and IP-address of the gateway (the IP-address of the server running the gateway) must be entered in the phone.

**Figure 3:**
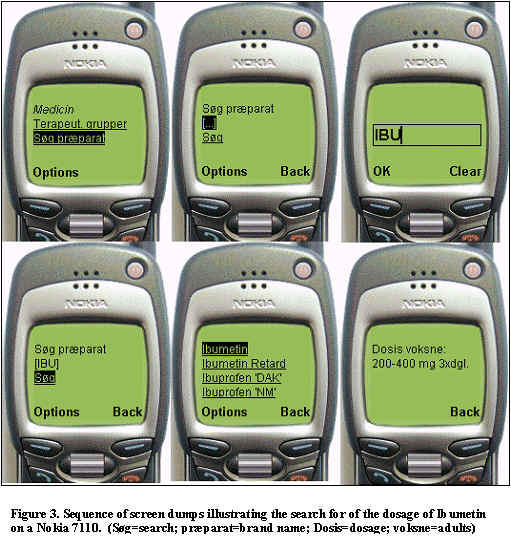
Sequence of screen dumps illustrating the search for of the dosage of Ibumetin on a Nokia 7110

## Results

A data driven interactive WAP-based pharmaceutical catalogue was established. Access to the individual brand names was available through free text search or by browsing the therapeutic groups. The application can be tested at http://hilist.au.dk/wap.php by using a public gateway or by using a WAP emulator on the www (Figure 3). Response time for accessing a new level in the catalogue hierarchy or completing a search was usually less than three seconds. Searching a brand name, which could be completed in only a few steps (Figure 3) in most cases was found to be faster than browsing the content hierarchy. The application was tested on Nokia 7110 and Ericsson R320s WAP phones. Several device-specific limitations were revealed. The display resolution is 95 x 45 pixels for the Nokia 7110 and 101 x 52 pixels for Ericsson R320s allowing four (Nokia) or five (Ericsson) lines of text to be displayed. The maximum amount of data per card (the maximum card size) was 1397 bytes for the Nokia and 3000 bytes for the Ericsson. These limitations must be considered when designing the WML pages (split data in a sequence of cards).

## Discussion

With the present project we have demonstrated that an open source freeware WAP gateway to a complex database can be established with information of clinical relevance. However, a number of practical and technological problems still have to be solved before WAP devices can effectively substitute or supplement other devices for processing clinical information. Because of the high energy transmitted while communicating with GSM phones, their use is still prohibited within many hospital wards and the security is under debate [7,8]. Yet there seem to be several solutions to this problem. Handheld WAP devices, using a comparable communication technology, but transmitting significantly less energy may be used. The development of medical electronic devices for use on hospital wards is towards protection of individual devices that allows use of regular GSM communication without interference. The small screen and relatively ineffective input tools of the WAP phone should be improved. The first steps towards speech control have been taken on some newer WAP phones. Further development in this direction will significantly improve usability [9]. Doctors may connect to databases and even call for data on a specific patient by use of speech control. Further, the present speech message technology found in, for instance, the Ericsson R320s could be further developed to allow functions that are traditionally found in dictaphones. This would allow the physician to edit and finish a full dictation before sending the note for entry into the patient record. This technology will offer many advantages compared with present technologies; for example the secretary will have the dictated note directly available without a risk of audiotapes being mislaid and possibly the speech message could be stored on a central server for temporary access by others before it has been entered into the patient record. Testing the use of WAP phones for information processing in a clinical ward was not part of the present project. However, this project has shown that even with the small screen and scrolling text, once connection to the server is established, it is possible to fetch text from the database with a speed that comes close to normal reading speed. Entering larger amounts of text, however, is time-consuming on a cellular phone keyboard so we conclude that for text input is a bigger problem than output. New technologies are constantly being developed in an extremely dynamic market for handheld communication devices. Bandwidth is increased using i.e. the GPRS or UTMS services in conjunction with Bluetooth and other local wireless communication technologies. Functions found in PDA devices are being incorporated into cellular phones. Technology however, needs to be adapted to the clinical reality before we can expect a widespread use by physicians.

## Abbreviations

Apache: is a freely available Web server that is distributed under an "open source" license. It runs on most UNIX-based operating systems
ASCII: American Standard Code for Information Interchange is the most common format for text files in computers and on the Internet. Bluetooth is a specification that describes how mobile phones, computers, and personal digital assistants can easily interconnect with each other and with home and business phones and computers using a short-range wireless connection Gateway is a network point that acts as an entrance to another network
GPRS: General Packet Radio Service is a packet-based wireless communication service that promises data rates from 56 up to 114 Kbps and continuous connection to the Internet for mobile phone and computer users
GSM: Global System for Mobile communication is the most widely used of the three digital wireless telephone technologies (TDMA, GSM, and CDMA). GSM digitizes and compresses data, then sends it down a channel with two other streams of user data, each in its own time slot. It operates at either the 900 MHz or 1800 MHz frequency band.
Http: Hypertext Transfer Protocol is the set of rules for exchanging files (text, graphic images, sound, video, and other multimedia files) on the World Wide Web
HTML: Hypertext Markup Language is the set of mark-up symbols or codes inserted in a file intended for display on a World Wide Web browser page.
ISP: An Internet Service Provider is a company that provides access to the Internet and other related services
IP: The Internet Protocol is the method or protocol by which data is sent from one computer to another on the Internet Linux is a UNIX-like operating system that was designed to provide personal computer users a free or very low-cost operating system comparable to traditional and usually more expensive UNIX systems
MIME: Multi-Purpose Internet Mail Extensions is an extension of the original Internet e-mail protocol that lets people use the protocol to exchange different kinds of data files on the Internet.
MySQL: is an open source relational database management system that uses Structured Query Language (SQL), for adding, accessing, and processing data in a database.
Perl: Practical Extraction and Reporting Language is a script programming language that is similar in syntax to the C language. It was invented by Larry Wall.
PDA: Personal Digital Assistant is a term for any small mobile hand-held device that provides computing and information storage and retrieval capabilities
PHP: is a script language and interpreter that is freely available and used primarily on Linux Web servers. The initials come from the earliest version of the program, which was called "Personal Home Page Tools"
SMS: Short Message Service is a service for sending messages of up to 160 characters to mobile phones that use Global System for Mobile (GSM) communication
UMTS: Universal Mobile Telecommunications System is a broadband, packet-based transmission of text, digitized voice, video, and multimedia at data rates up to and possibly higher than 2 megabits per second (Mbps)
URL: Uniform Resource Locator is the address of a file (resource) accessible on the Internet
WML: Wireless Markup Language, allows the text portions of Web pages to be presented on cellular telephone and personal digital assistants (PDA) via wireless access.
